# The impact of the pupil size artifact on pupil-based eye-tracking data in reading tasks: Assessment and compensation

**DOI:** 10.3758/s13428-025-02912-y

**Published:** 2025-12-19

**Authors:** Wolf Culemann, Angela Heine, Ignace T. C. Hooge

**Affiliations:** 1 Institute of Psychology, University of Duisburg-Essen, Universitätsstr. 2, Essen, 45141 Germany; 2 Experimental Psychology, Helmholtz Institute, Utrecht University, Utrecht, The Netherlands

**Keywords:** Eye tracking, Pupil size artifact, Reading research, Data quality

## Abstract

Eye tracking in reading research requires high spatial accuracy due to small, densely arranged areas of interest. However, despite widespread use of pupil-based eye trackers in reading studies, a major source of systematic inaccuracy remains largely unaddressed: the pupil size artifact (PSA) – apparent gaze shift in fact caused by pupil dilation even when the eye remains stationary. Using pupillary light reflex manipulations and reading tasks under controlled luminance, we quantified gaze inaccuracy due to the PSA and compared correction methods. We observed systematic apparent gaze shift of up to 2$^{\circ }$ as pupil sizes varied from 2 to 6 mm. Horizontal PSA showed contralateral patterns (median slopes: 0.38$^{\circ }$/mm), while vertical PSA increased with pupil size (up to 0.86$^{\circ }$/mm for larger pupils). Even under constant luminance, pupil size varied substantially during reading (median 95% ranges 0.78–1.38 mm). We compared two correction approaches: line assignment (standard in reading research) and PSA recalibration (modeling pupil-size-induced apparent gaze shift across the screen). Both methods effectively corrected vertical apparent gaze shift, showing similar average correction offsets and sensitivity of correction to pupil size, suggesting that line assignment implicitly compensates for vertical PSA effects. However, only PSA recalibration addressed horizontal apparent gaze shift, reducing overall gaze shift by over 50% and improving the performance of line assignment algorithms. Our findings underscore the importance of accounting for PSA in reading research. We offer practical recommendations for improving gaze accuracy in eye-tracking reading studies.

**Supplementary Information:**

The online version contains supplementary material available at 10.3758/s13428-025-02912-y.

## Introduction

Eye tracking technology has become an invaluable tool in reading research, allowing for insights into cognitive processes through the analysis of eye movements (Hessels et al., 2025; Rayner, 1998). However, reading research places high demands on the spatial accuracy of eye tracking data, as areas of interest – typically individual words – are often small and densely arranged. Many widely used metrics (e.g., word frequency effect, landing position, re-reading duration) depend on accurately assigning fixations to words and are therefore highly sensitive to spatial error (Holmqvist et al., 2012, see inaccuracy).

Most modern reading studies rely on video-based eye trackers, which estimate gaze position using the pupil center and corneal reflection. While widely adopted, these systems are susceptible to inaccuracies arising from head movement, imperfect calibration, and spatial distortions across the screen – often worsening near screen edges (Carr et al., 2022; Holmqvist et al., 2012; Niehorster et al., 2018). Reading researchers are well aware of these accuracy challenges and commonly use post hoc correction methods to improve gaze accuracy (Carr et al., 2022). Common approaches include enlarging areas of interest around words or assigning fixations to their most probable word, either through manual adjustment, a process that is both time-consuming and subject to inconsistency (Carr et al., 2022; Cohen, 2013; Mercier et al., 2024), or using algorithmic approaches (Carr et al., 2022; Cohen, 2013; Culemann et al., 2024; Glandorf and Schroeder, 2021; Mercier et al., 2024; Schroeder, 2019).

Despite these efforts, one systematic source of gaze inaccuracy has received surprisingly little attention in reading contexts: the pupil size artifact (PSA). When the pupil dilates or constricts, pupil-based eye tracking systems can report an apparent shift in gaze even when the eye does not rotate (Choe et al., 2016; Drewes et al., 2014; Hooge et al., 2019, 2021, 2025; Wildenmann & Schaeffel, 2013; Wyatt, 2010). This apparent gaze shift can be large, up to 2.5$^{\circ }$ or even 5$^{\circ }$ of visual angle (Drewes et al., 2014), can differ between the eyes and occur in both horizontal and vertical directions (Choe et al., 2016; Hooge et al., 2021; Jaschinski, 2016; Wyatt, 2010). Importantly, the PSA appears to be idiosyncratic, but stable over time within individuals, which makes it possible to compensate for it (Drewes et al., 2014; Hooge et al., 2021, 2025; Ivanov & Blanche, 2011).

Two well-documented factors contribute to the PSA (Choe et al., 2016). First, physiological pupil decentration: as the iris sphincter and dilator muscles change aperture, the center of the pupil can shift within the eyeball (Wyatt, 2010). Second, viewing geometry and optics: The apparent center of the pupil in the camera image depends on the angle between the viewer’s line of sight and the tracking camera. Off-axis viewing leads to foreshortening effects (i.e. while the pupil appears reasonably circular when viewed from the front, it appears elliptical when viewed from the side) and additional corneal refraction effects, so that the size and direction of the PSA depend on the relative viewing angle to the camera and thus on the details of the setup (Drewes et al., 2014; Hooge et al., 2021; Wildenmann & Schaeffel, 2013). As a consequence, spatial patterns of the PSA across the display are expected to be setup specific: different camera placements (e.g., centered vs. offset to one side), and screen size can yield different PSA patterns (Hooge et al., 2021). This setup dependence can also provide a principled explanation for seemingly inconsistent patterns across laboratories.

The PSA is particularly relevant to reading research for at least two key reasons. First, pupil size is not only influenced by luminance but also by emotional arousal, task difficulty, mind wandering, and word surprisal (Franklin et al., 2013; Frank & Thompson, 2012; Mathôt, 2018). Even in carefully controlled lighting conditions, pupil size can fluctuate significantly – by as much as 2 mm (Hooge et al., 2025). As a result, changes in pupil size can induce apparent gaze shift that systematically co-vary with experimental conditions (Jaschinski, 2016), potentially mimicking or masking the very cognitive effects researchers aim to detect.

Second, reading tasks require high spatial accuracy in vertical and horizontal dimensions: even small gaze inaccuracies can result in fixations being misattributed to the wrong word or line, potentially influencing the power to detect effects of key reading metrics, such as word frequency, predictability, word length, landing position or launch site, or even distorting them. While, many correction methods focus on vertical correction only (e.g., line assignment, Carr et al., 2022; Mercier et al., 2024; Schroeder, 2019), horizontal gaze inaccuracies remain a critical concern and are often unaddressed (Mercier et al., 2024). Interestingly, Carr et al. , (2022, p. 288) describe vertical drift as “occur[ring] quite unpredictably” with underlying causes that “can be difficult to control for, even in a laboratory setting.”

Should the PSA represent a substantial component of these putatively unpredictable inaccuracies, accounting for pupil size changes would transform seemingly random variations into systematic and correctable patterns, potentially leading to improved correction methods. Notably, PSA-specific correction methods based on additional calibration that take into account the size of the pupil have already improved gaze accuracy in non-reading contexts (Choe et al., 2016; Drewes et al., 2014; Hooge et al., 2025; Jaschinski, 2016), suggesting their potential value in reading research as well.

To address this gap, the present study systematically investigates the PSA using both dedicated calibration procedures with known target positions and a reading task. We approach this by first establishing the magnitude and individual variability of the PSA, then assessing its practical relevance during typical reading conditions, developing and validating a dedicated PSA recalibration method, and finally comparing this approach with the established practice of line assignment in the reading field. We address the following research questions:

1. *What is the extent of spatial inaccuracy due to pupil size changes?* We quantify the magnitude and individual variability of pupil-size-induced apparent gaze shift for each participant across screen dimensions and both eyes, using calibration data with known target positions from dedicated PSA calibration procedures. This provides essential baseline data for interpreting existing research and informing future study designs.
2. *What magnitude of pupil-size-induced apparent gaze shift should researchers expect during reading?* We assess pupil size variation during reading under controlled luminance conditions to determine the practical relevance of PSA effects. This will establish whether PSA represents a negligible source of error (if variation is minimal) or a significant concern requiring correction (if variation is substantial).
3. *How well can a dedicated PSA recalibration method correct for pupil-size-induced apparent gaze shift?* To address the putative PSA effects identified above, we implement and test a correction approach that explicitly models pupil-size-induced apparent gaze shift across the screen using an additional calibration procedure, building on previous work (Choe et al., 2016; Drewes et al., 2014; Hooge et al., 2025; Ivanov & Blanche, 2011). We evaluate its performance using validation data with known target positions.
4. *How do the corrective offsets applied by a dedicated PSA recalibration method compare to those applied by the commonly used line assignment approach during reading?* To assess implications for reading research practices, we compare the corrective offsets applied by a PSA correction method with those applied by line assignment, which assumes that fixations predominantly target text lines and is widely accepted in reading research. Do both methods apply corrections of similar magnitude and show similar sensitivity to pupil size changes (i.e., do corrections change with pupil size)?

## Methods

### Overview of experimental approach

To investigate our research questions, we designed an experiment to estimate apparent gaze shifts caused by pupil size changes and to capture gaze and pupil size behavior under luminance-controlled reading conditions. The study consists of two experimental manipulations:

1. *Pupillary light reflex (PLR) phases:* In this manipulation, pupil size changes are induced at different screen locations by changing the luminance through background color switches. We measure the resulting apparent gaze shift (horizontal and vertical offsets from known target positions) and use these measurements to construct a pupil-size correction model. Each phase consists of one of the two conditions, which we refer to as calibration and validation. Note that these are distinct from the initial eye tracker calibration. The calibration condition is used to assess the magnitude of the PSA for our setup (Research Question 1), as well as fitting a dedicated PSA recalibration model (Research Question 3). The validation condition is used to evaluate how well this PSA recalibration model generalizes to new timepoints and screen locations (Research Question 3).
2. *Reading blocks:* Participants read texts under three luminance conditions (bright, medium, dark) with constant background brightness within each condition, designed to reflect commonly used background luminance conditions in lab-based reading studies. These reading blocks allow us to examine how pupil-size-induced apparent gaze shift behaves under varied but realistic experimental conditions. Apparent gaze shift is quantified as vertical offsets from expected text line positions and used to evaluate effectiveness of line assignment and the dedicated PSA correction model. The reading blocks are used to assess the extent of pupil size changes during reading (Research Question 2), and to compare the corrections by PSA recalibration to that of the line assignment approach on reading data (Research Question 4).

### Participants

Thirty-three participants (age 24.15 years, range 18–38; gender 27 female, five male, one other) completed the study. All participants had normal vision, did not require visual aids, and did not wear mascara during the experiment. The majority were university students who received course credits for their participation. Ethical approval was obtained from the University of Duisburg-Essen’s ethics committee (EA-PSY22/24/18112024).

### Apparatus

Gaze position and pupil size signals were recorded binocularly at 1000 Hz using a desktop-mounted EyeLink 1000 Plus eye tracker (SR-Research), using the ellipse pupil detection model and the heuristic filters turned off. Participants were seated 93 cm from an ASUS VG248QE monitor (resolution 1920 $\times$ 1080 pixels; dimensions 53.1 $\times$ 29.1 cm; refresh rate 120 Hz). Head position was stabilized with a chin and forehead rest, and the room was dimly lit using two indirect lamps to maintain constant ambient illumination. The eye tracker was initially calibrated using EyeLink’s standard 13-point procedure on a medium gray background (gray[110], where numbers indicate gray levels on an 8-bit scale from 0=black to 255=white), with a black ABC target (Thaler et al., 2013) with an outer diameter of 0.6$^{\circ }$. Calibration was accepted only if validation showed offsets below 1$^{\circ }$ for each validation point and for both eyes, as verified by EyeLink’s built-in validation procedure.

### Procedure

**Fig. 1:**
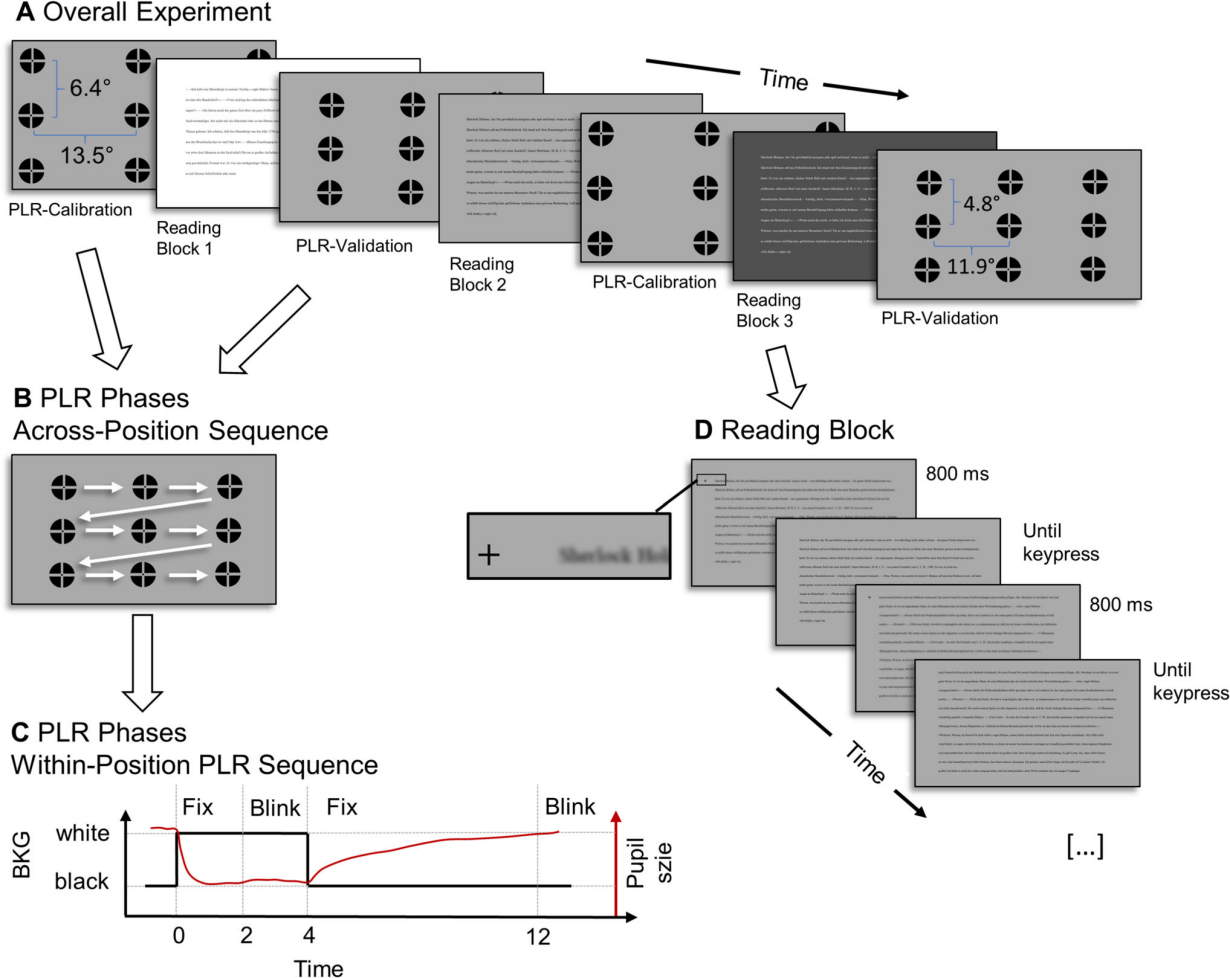
Overview of the experimental protocol. The experiment consists of alternating reading blocks and pupillary light reflex (PLR) phases. **A** Overall experiment design: Participants read three chapters under different luminance conditions (bright, medium, dark), with luminance-condition–chapter assignment randomized per participant. PLR phases were interleaved between reading blocks. PLR Calibration used more peripherally located targets and PLR validation used more centrally located targets. **B** Targets in the PLR phases appeared in a fixed across-position sequence (left to right, top to bottom). **C** Within-position sequence: Each target followed a bright-dark transition: The screen was initially black, then turned white for 2 s, followed by an interleaved pulsing sequence that allowed participants to blink and prepared them for the fixation period, where steady fixation and minimal blinking were required. Next, the screen switched back to black for 8 s, after which a final pulsing sequence occurred before moving to the next target. **D** Reading block procedure: Each page began with a fixation cross positioned to the left of the first text line, during which the text remained blurred. The full reading page was then displayed until the participant pressed the spacebar to proceed. Note: The *gray values* and the relative size of the ABC targets in this figure differ from those used in the actual experiment and have been adjusted for presentation purposes

Each participant completed four PLR phases (two calibration, two validation) interleaved with three reading blocks under different luminance conditions (Fig. 1A).

#### PLR phases

To systematically induce changes in pupil size, we used the pupillary light reflex (PLR) – the automatic constriction and dilation of the pupil in response to changes in luminance. Participants fixated ABC targets (Thaler et al., 2013) (outer diameter 0.6$^{\circ }$) at known screen locations while luminance changes due to a switch in background color triggered systematic pupil size changes. This was done with both eyes open, i.e., no separate monocular runs. We used two distinct nine-point target grids: one peripheral (± 13.5$^{\circ }$ horizontal, ± 6.4$^{\circ }$ vertical) for PLR calibration, and one more central (± 11.9$^{\circ }$ horizontal, ± 4.8$^{\circ }$ vertical) for PLR validation. PLR validation used a different set of target positions to avoid circularity and to assess spatial interpolation of the mapping learned during PLR calibration – i.e., performance at previously unseen (*x*, *y*) positions. Targets appeared in a fixed sequence from left to right, top to bottom (Fig. 1B), to help participants anticipate where to look next and to mimic the gaze sequence during reading. A PLR phase lasted approximately 2 min in total.

At each target location, participants experienced a bright-dark transition sequence: the initially black screen was followed by stable fixation periods of 2 s on a white screen and 8 s on a black screen. The timing of this sequence follows Hooge et al. (2025), and takes into account that pupil dilation takes more time than constriction. Between stable fixation periods, a pulsing cue was shown to prepare for the required steady fixations and to explicitly allow participants to blink to avoid data loss during the stable fixation periods (Fig. 1C). This pulsing cue consisted of an expanding and contracting central dot presented at the end of the white phase, before the screen turned black again. Note that our analyses rely on the resulting pupil size changes, not the specific background luminance or the background transition timing. For this reason, the specific monitor luminance or TFT panel transition time does not affect our analyses.

#### Reading blocks

Between PLR phases, participants read chapters from “The Hound of the Baskervilles” (in German) under three different luminance conditions: bright (gray[255/40]), medium (110/0), and dark (0/150). The three chapters (containing 9, 15, and 11 pages respectively) were presented in different luminance conditions, with the luminance-condition–chapter assignment randomized for each participant. Text appeared in 24-point Times New Roman, with characters subtending on average 0.16$^{\circ }$ in width and 0.37$^{\circ }$ in height. The text area extended 12.75$^{\circ }$ horizontally and 7$^{\circ }$ vertically from screen center, with 1.25$^{\circ }$ line spacing. Each page began with an 800-ms fixation cross (1.85$^{\circ }$ left of the first text line) while text remained blurred, followed by clear text presentation until participants pressed the spacebar to continue (Fig. 1D). Pages contained approximately 250 words each to maintain consistent reading blocks.

### Eye-tracking data processing

The eye-tracking data underwent three main processing steps: (1) blink removal, (2) pupil signal quality assessment via visual inspection, and (3) unit conversion.

Fixations were identified by the EyeLink’s velocity-based event detection algorithm (saccade detection thresholds: 30$^{\circ }$/s and 9500$^{\circ }$/s$^{2}$). Very short ($<80~ms$) and very long ($>800~ms$) fixations were excluded, following common procedures in eye tracking reading studies (e.g., Ashby et al., 2012; Wang et al., 2024). Start and end times of fixations were used for blink removal in reading data, and fixation positions were used for comparing line assignment and PSA recalibration (see Section Correction methods). Note, how analyses of PSA slopes and the PSA recalibration were conducted on single samples – that is, raw gaze and pupil size data points recorded at 1000 Hz – as pupil-size-induced apparent gaze shift can occur on short time scales (Choe et al., 2016).

#### Blink removal

For PLR phases, potential blink periods were first identified using a moving 60-s window to detect sudden pupil size deviations exceeding ±20 % from the local average, allowing detection of partial blinks not captured through data loss alone (Culemann et al., 2023). These detections were then refined with the algorithm by Hershman et al. (2018) to account for gradual pupil changes surrounding blinks. Consecutive blink events with inter-blink intervals (from offset to onset) shorter than 100 ms were merged into single blink episodes. For reading blocks, blink removal was conducted by removing data outside of fixations.

#### Pupil signal quality assessment (Visual Inspection)

Visual inspection of fixation scanpaths and pupil size time series revealed systematic tracking artifacts, visible as large spikes in the pupil size signal, occurring predominantly when pupil diameters exceeded 6–7 mm. These artifacts were likely caused by pupil boundary detection issues due to eyelash interference in the pupil thresholding process of the EyeLink system. Consequently, we excluded three subjects who exhibited these artifacts in both medium and dark luminance conditions from all analyses. Four additional subjects who showed these artifacts only in the dark condition were retained for analyses of the bright and medium conditions.

#### Unit conversion

The EyeLink 1000 Plus pupil signal was converted from pupil area in arbitrary units (SR Research, 2010) to pupil diameter in millimeters using offline calibration, using pupil-only recordings of printed black dots of known diameter to derive setup-specific conversion factors (Mathôt and Vilotijević, 2022; SR Research Support, 2025). Gaze coordinates were transformed from pixels to degrees relative to the screen center.

### Quantitative data quality assessment

Following preprocessing, we conducted a quantitative assessment of data quality as a standard and transparent reporting practice. This included calculating signal precision and quantifying data loss rates for both PLR phases and reading blocks.

Signal precision was assessed using the root mean square of sample-to-sample distances (RMS-S2S), following the sliding window approach described by Hooge et al. (2018). A window size of 100 ms (100 samples) was applied to both gaze position and pupil size signals. For the PLR phases, the data showed a mean RMS-S2S of 0.0118$^{\circ }$ (SD =0.0050$^{\circ }$) for gaze position, and 0.0014 mm (SD = 0.0005 mm) for pupil size. Mean data loss during these phases was 1.24% (SD = 5.94%). For the reading blocks, mean RMS-S2S values were 0.0164$^{\circ }$ (SD = 0.0042$^{\circ }$) for gaze position and 0.0015 mm (SD = 0.0003 mm) for pupil size. Data loss in these blocks averaged 1.95% (SD = 3.29%).

### Quantification of the pupil size artifact

To quantify the relationship between apparent gaze shift and simultaneously measured pupil size using PLR calibration data (Jaschinski, 2016), we use the *PSA slope*. The PSA slope indicates how much the gaze position shifts per millimeter change in pupil size and is used to assess the magnitude and individual variability of the PSA (Research Question 1). For aggregated statistical analyses across participants, we use the absolute PSA slope to focus on the magnitude of this relationship regardless of direction, since PSA slopes have been shown to vary in their direction across screen positions and between individuals (Drewes et al., 2014; Hooge et al., 2021).

### Correction methods

We compared two approaches that correct for potential inaccuracies in gaze position during reading. We first evaluated the performance of a dedicated PSA correction method using PLR Validation data with known target positions (Research Question 3), then compared this approach to line assignment during reading (Research Question 4).

#### PSA recalibration

Conceptually, PSA recalibration creates a three-dimensional lookup table, similar to that of Drewes et al. (2014), in which each combination of reported gaze position and pupil size is associated with an offset to correct for pupil-size-induced apparent gaze shift. In other words, the method establishes a mapping function that explicitly incorporates both gaze position and pupil size to estimate and correct for systematic shifts in reported gaze location. We fitted this mapping using the PLR Calibration data. This is analogous to standard eye-tracker calibration, but it explicitly takes pupil size into account and corrects – already calibrated – measured gaze position on screen (potentially affected by PSA) to an estimate of the true gaze position. The mapping function is based on fitting the relationship between pupil size and apparent gaze shift, where apparent gaze shift was operationalized as the offset between the measured gaze signal and the known target coordinates that participants were instructed to fixate.

Our mapping employs a two-stage approach: First, for each target position, we fit 2nd-order polynomial regressions relating pupil size to apparent gaze shift in both horizontal and vertical directions. Second, we fit 2D polynomials across screen positions to model how these local coefficients vary continuously across the screen, which allows prediction of pupil-size-induced corrective offsets for any position on screen. Note how this gives us a prediction of the apparent gaze shift due to the PSA for a true position on screen, but when correcting a measured gaze position signal, the true position on screen is unknown. To address this inverse mapping problem (correcting gaze positions that already contain offsets), we implemented a comprehensive lookup table approach, where we store the mappings of predicted gaze position – containing offsets – to the corrective offset. We generated a dense 3D grid of positions and pupil sizes (0.1$^{\circ }$ spatial steps, 0.1 mm pupil size steps), calculated the expected offsets at each point, and stored these mappings. During correction, we identify the appropriate offset for any measured gaze position and pupil size and subtract it to recover the corrected position. The model handles values outside the measured range through nearest-neighbor extrapolation. For more detailed information about the implementation, we refer the reader to the code implementation in the corresponding OSF repository (see Data and Code Availability for the link).

#### Line assignment

While PSA recalibration provides a dedicated approach for correcting pupil-size-induced apparent gaze shift, line assignment represents the most commonly used correction method in multi-line reading research. This approach maps each fixation during reading to its most probable text line, allowing calculation of apparent vertical gaze shift as the distance between each gaze sample and its corresponding text line position.

Line assignment can be conducted through various appro-aches, including manual correction and different algorithms with varying strengths and weaknesses (Al Madi, 2025; Carr et al., 2022; Cohen, 2013; Mercier et al., 2024). To ensure our comparison represents effective line assignment practices, we implemented a robust semi-automated approach. We achieved fixation-to-text-line assignment through a semi-automated process in Eyeflow Studio (Culemann & Heine, 2024), consisting of four stages: (1) initial algorithmic correction applied separately for each eye and text chapter using the method by Culemann et al. (2024), (2) averaging fixations from both eyes, (3) fixation-to-line assignment using the “chain” algorithm (Schroeder, 2019), and (4) manual supervision with correction of misaligned fixations. Once line assignments were finalized using the averaged fixations, we mapped these assignments back to each eye’s fixation and raw sample data.

This semi-automated process is likely more robust than single-algorithm line assignment methods, which are susceptible to misassignments from various sources (Carr et al., 2022). Similar to the gold standard of manual correction by multiple expert coders (Carr et al., 2022), our approach represents the upper limit of vertical correction effectiveness achievable through current line assignment methodologies, providing a strong benchmark for comparison with PSA recalibration.

#### Comparison of correction methods

To assess implications for reading research practices, we compared the corrective offsets applied by PSA recalibration to those applied by line assignment. Since line assignment only provides corrections in the vertical dimension, our comparison focused on vertical corrective offsets. Since the reading data lack known ground truth target positions for direct accuracy assessment, we focus on comparing the magnitude and patterns of corrections applied by each method. Differences in correction magnitude may indicate the presence and extent of PSA effects that each method addresses. We calculated metrics to characterize correction magnitude and pupil size sensitivity (correction change with pupil size) for each method, analyzed using linear mixed-effects models implemented in R (version 4.3.2, R Core Team, 2021) using the lme4 package (version 1.1.35.1, Bates et al., 2015).

## Results

### Magnitude and individual variability of the PSA

**Fig. 2:**
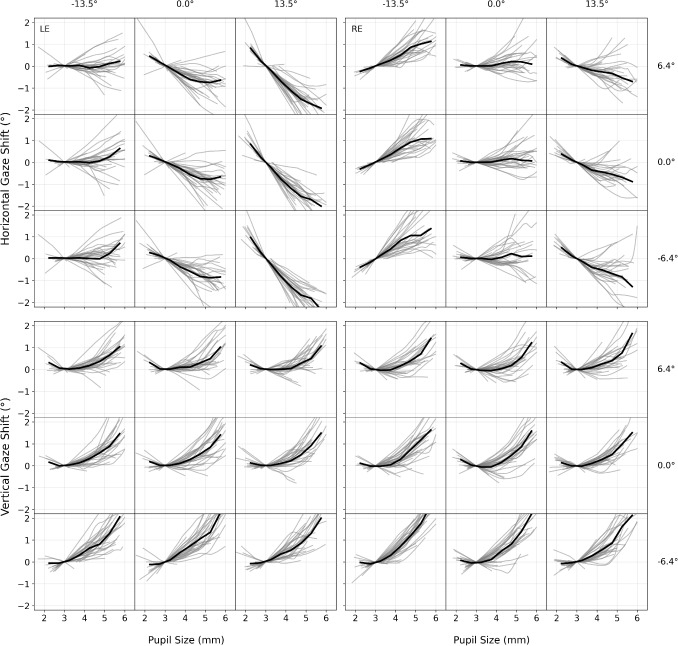
Apparent gaze shift across pupil sizes for nine screen positions and all subjects. Each subpanel shows LOWESS curves representing the relationship between pupil size and apparent gaze shift from targets at different screen locations (± 13.5$^{\circ }$ horizontal, ± 6.4$^{\circ }$ vertical). *Left panels* show left eye data; *right panels* show right eye data. *Top panels* display horizontal gaze offset; *bottom panels* show vertical gaze offset. *Gray lines* represent individual subject curves (LOWESS smoothing with 1-mm constant window width); *black lines* show group averages. All curves are normalized to zero at 3-mm pupil size to allow comparison independent of initial calibration conditions

To quantify the PSA for our subjects and setup, we analyzed apparent gaze shift from known target positions in the PLR Calibration blocks. For visualization of the apparent gaze shift across the full range of pupil sizes, we used LOWESS smoothing (Cleveland, 1979) with an adaptive span parameter maintaining a constant 1-mm pupil diameter window. Figure 2 shows LOWESS curves for individual subjects (*gray*) and group averages (*black*) across the nine screen positions, and both eyes. All curves are normalized to zero at 3-mm pupil size to allow comparison independent of initial eye-tracker calibration conditions (see Section The relevance of pupil size during eye tracker calibration).

The visualization reveals high intersubject differences, but also substantial PSA magnitudes across participants, with average apparent gaze shifts approaching 2$^{\circ }$ as pupil size varies between 2 and 6 mm. These findings replicate and extend previous reports (Choe et al., 2016; Drewes et al., 2014; Hooge et al., 2021; Wildenmann & Schaeffel, 2013; Wyatt, 2010) by comprehensively visualizing the PSA across pupil size, screen positions, and both eyes. We observed systematic spatial differences across eyes and positions. Horizontal shifts showed a contralateral pattern – larger (signed) slopes occur when the target is on the side opposite to the measured eye – mirrored between eyes with slight asymmetries. Vertical slopes were larger toward the bottom of the screen and more similar between eyes. Horizontal slopes were relatively stable across pupil-size intervals, whereas vertical slopes tended to increase with pupil size.

**Fig. 3:**
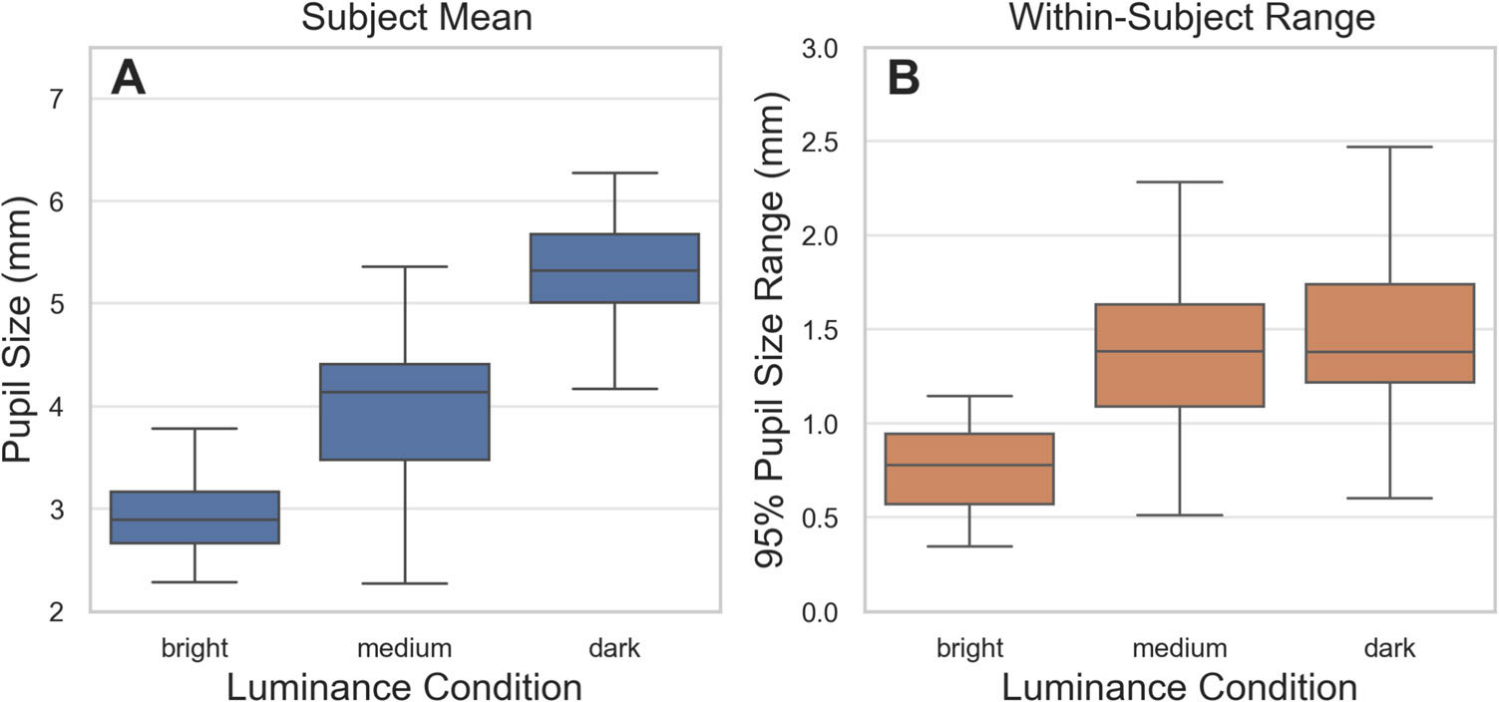
Mean pupil size and within-subject pupil size range during reading under different luminance conditions across all subjects. **A** Subject mean – mean pupil diameters for each subject during reading across luminance conditions (bright, medium, dark). Pupil size systematically increases with decreasing luminance, a finding important for PSA considerations as larger pupils typically show stronger apparent gaze shift effects. **B** Within-subject range – per-subject pupil size ranges within luminance conditions (95% range, the difference between 2.5th and 97.5th percentiles, while reading a chapter of 9–15 pages under constant luminance). Even under controlled lighting, substantial pupil size changes occur (median ranges 0.78–1.38 mm), with both smaller ranges on average and reduced between-subject differences in bright conditions (visible in the more compact box and whiskers). *Error bars* represent the interquartile range, with whiskers extending to 1.5 $\times$ IQR

### Pupil size and apparent gaze shift due to the PSA during reading

To determine whether PSA effects are likely to arise even in luminance-controlled experimental designs, we examined how much pupil size changed during reading under constant luminance conditions. Since pupil-size-induced apparent gaze shift is a function of pupil size changes, understanding the magnitude of these changes during reading is critical for assessing PSA impact.

Figure 3 presents pupil sizes across bright, medium, and dark luminance conditions. Median pupil sizes increased systematically with decreasing luminance 2.89 mm (95% range 2.41–3.66 mm) in bright, 4.14 mm (95% range 2.86–5.78 mm) in medium, and 5.32 mm (95% range 3.48–6.62 mm) in dark conditions (Fig. 3A). This finding is particularly relevant because larger pupil sizes tend to exhibit larger PSA slopes, especially for vertical apparent gaze shift.

Even more importantly, pupil sizes varied substantially within each subject during reading, even when luminance remained constant. Figure 3B shows the per-subject pupil size range within luminance conditions (each containing 9–15 pages of text). The median 95% range of pupil diameter within luminance conditions was 0.78 mm (95% range across participants 0.38–1.14 mm) in bright, 1.38 mm (95% range across participants 0.57–2.28 mm) in medium, and 1.38 mm (95% range across participants 0.73–2.44 mm) in dark conditions. Notably, these pupil size changes occur continuously throughout reading: analysis of individual text pages revealed similar patterns, with median per-subject ranges of 0.63 mm (95% range across participants 0.32–1.23 mm) in bright, 1.06 mm (95% range across participants 0.52–2.04 mm) in medium, and 0.98 mm (95% range across participants 0.49–2.14 mm) in dark conditions. The similarity between page-level and chapter-level within-luminance ranges indicates that pupil size fluctuations are an ongoing process throughout reading.

Three important patterns emerge from these data: (1) substantial pupil size changes occur within subjects even under constant luminance, regardless of the analysis timescale; (2) the average pupil size range was smaller in bright versus medium/dark conditions; and (3) the between-subject differences in pupil size ranges (visible in the size of the boxes and whiskers) were also reduced in bright conditions, suggesting more consistent measurement conditions across participants.

Both median pupil size and pupil-size changes increased as luminance decreased. This pattern aligns with findings from Drewes et al. (2014), who reported that pupil dynamics increase in magnitude with decreasing luminance. The bright condition consistently showed both smaller pupil sizes and smaller ranges of change compared to medium and dark conditions.

To evaluate the practical relevance of the PSA for reading tasks, we estimated the apparent gaze shift expected during reading from the product of the PSA slope ($^{\circ }/\text {mm}$) and the amount of pupil-size change (mm). PSA slopes were taken from the LOWESS-based PSA curves (Fig. 2) and, for each subject and condition, we used the mean slope across the pupil-size range that actually occurred in that condition. While these estimates are approximate and intended to give an order-of-magnitude indication rather than accurate predictions, they help contextualize the potential impact of PSA in typical reading scenarios.

For each subject, eye, axis (horizontal/vertical), and screen region, we multiplied the PSA slope by the observed change in pupil size. Here, “PSA slope” refers to the average slope of the apparent gaze shift across the pupil size range observed under the respective conditions. We report the magnitudes (absolute values) and then average across subjects, eyes, and regions. We considered two practically relevant situations. (i) *Within-luminance*: Fluctuations under constant luminance, using each subject’s 95% pupil-diameter range (2.5th–97.5th percentiles) within a luminance condition (i.e., reading a text chapter). (ii) *Between-luminance*: Baseline shifts between luminance conditions, using per-subject differences in median pupil diameter (e.g., Bright$\rightarrow$Medium, Medium$\rightarrow$Dark). The latter also approximates what may happen if calibration and experiment differ in baseline pupil size, e.g., due to luminance or arousal.

**Table 1:** Estimated mean absolute apparent gaze shift magnitude during reading (in $^{\circ }$) caused by changes in pupil size

Condition	Horizontal	Vertical
Within-luminance		
Bright	0.39$^{\circ }$	0.24$^{\circ }$
Medium	0.58$^{\circ }$	0.69$^{\circ }$
Dark	0.64$^{\circ }$	1.03$^{\circ }$
Between-luminance		
Bright vs. Medium	0.51$^{\circ }$	0.50$^{\circ }$
Medium vs. Dark	0.49$^{\circ }$	0.66$^{\circ }$

Values are estimated per subject as $|\text {PSA slope}|\times |\Delta \text {pupil}|$ and then averaged across subjects, eyes, and screen regions. PSA slopes ($^{\circ }/\text {mm}$) are the average local slopes from the LOWESS-based PSA curves within the pupil-size range relevant to each condition. Within-luminance $\Delta \text {pupil}$ is the per-subject 95% range (2.5th–97.5th percentiles) under constant luminance (reading a text chapter); Between-luminance $\Delta \text {pupil}$ is the per-subject difference in median pupil diameter between luminance conditions (Bright vs. Medium, Medium vs. Dark). Horizontal and vertical dimensions were computed separately

Table 1 shows that the average apparent gaze shift magnitudes increased with decreasing luminance, for both within-luminance and between-luminance. Within luminance, estimated mean absolute apparent gaze shift reached from 0.39$^{\circ }$ horizontally and 0.24$^{\circ }$ vertically in bright conditions to 0.64$^{\circ }$ horizontally and 1.03$^{\circ }$ vertically in dark conditions. The estimates for the apparent gaze shift between luminance conditions (between bright and medium, and medium and dark) were on the order of 0.49$^{\circ }$ – 0.66$^{\circ }$. These averages, however, mask pronounced differences by screen position and eye. A few examples illustrate the range: for horizontal apparent gaze shift estimates within medium luminance, the left eye reached 1.17$^{\circ }$ for the right screen region, but 0.33$^{\circ }$ for the left screen region. Vertical apparent gaze shift estimates within medium luminance reached 0.84$^{\circ }$ for the bottom screen region, but 0.54$^{\circ }$ for the top screen region and were very similar for both eyes.

**Fig. 4:**
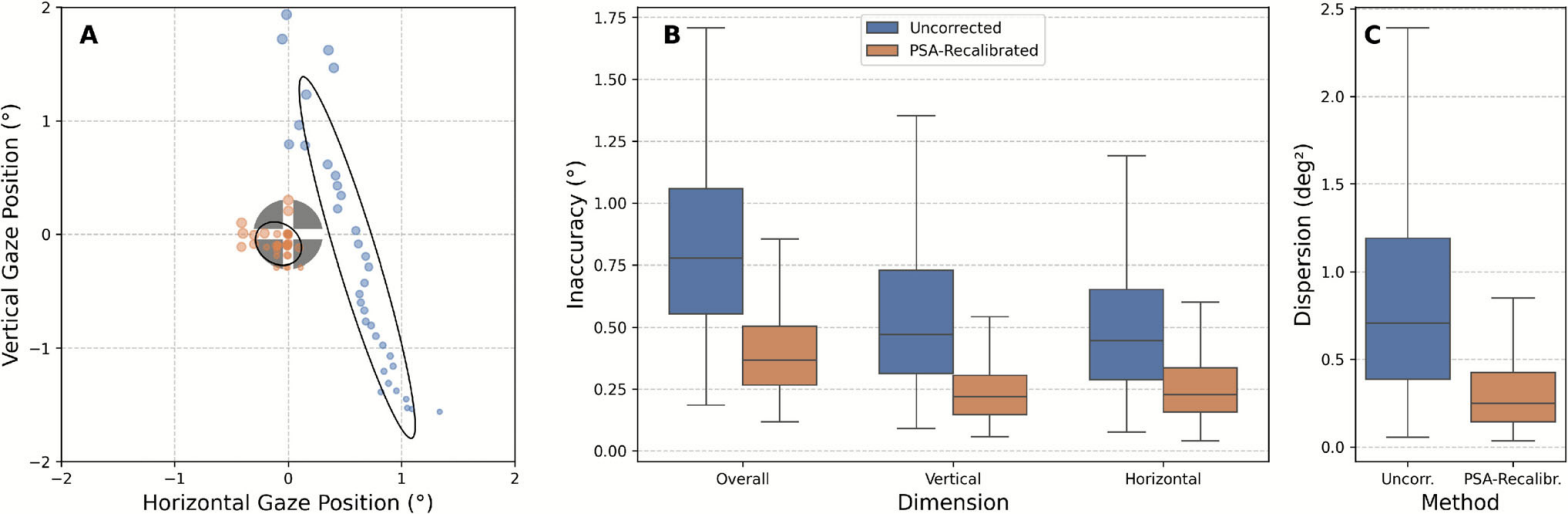
How effective is PSA recalibration in reducing pupil-size-induced apparent gaze shift during PLR validation? **A** Example from one subject (left eye) during a single prolonged fixation on the central target. Each *dot* is the median gaze position computed from all gaze samples – within this fixation – whose pupil diameter falls within a 0.1-mm bin (*dot area* encodes the bin’s median pupil diameter). Luminance changes resulted in systematic pupil size changes during this fixation. Overlaid 68% bivariate contour ellipse areas (BCEA) show the dispersion across these per-bin median positions. The *gray circle* represents the target (0.6$^{\circ }$ diameter). *Blue* before PSA recalibration; *orange* after. **B** Absolute offset (Euclidean distance between the median gaze position and the target) before vs. after PSA recalibration across all validation targets, eyes, and subjects. Values show how far median gaze positions deviate from target locations. **C** Gaze dispersion (68% BCEA of per-bin median positions) before vs. after. PSA recalibration reduces absolute offsets by a median of 50.6% (IQR 32.3–65.5%) and dispersion by a median of 63.0% (IQR 43.7–74.7%). *Error bars* represent interquartile range, with whiskers extending to 1.5 $\times$ IQR

Since estimates of apparent gaze shift due to pupil size changes within and between luminance conditions are naturally related (they depend on the same PSA slopes), a more differentiated picture also emerges for between-luminance apparent gaze shift estimates when considering the values for different screen positions and eyes. For example, between luminance conditions, horizontal apparent gaze shift estimates for the left eye due to pupil size changes from bright to medium were 1.05$^{\circ }$ for the right screen region as opposed to 0.24$^{\circ }$ for the left screen region. Vertical apparent gaze shift estimates for the left eye due to pupil size changes from bright to medium reached 0.70$^{\circ }$ for the bottom screen region as opposed to 0.36$^{\circ }$ for the top screen region. For a more detailed overview of the estimated magnitude of the apparent gaze shift during reading for different screen positions and eyes, please see the tables in the Appendix A. These patterns reflect the observed contralateral horizontal pattern of PSA slopes and the stronger vertical PSA slopes toward the bottom of the screen.

To relate these magnitudes to reading units, our study design used an average of 0.16$^{\circ }$/character and 1.25$^{\circ }$ line spacing. Thus, an estimated within-luminance horizontal inaccuracy due to pupil size changes in medium luminance (0.58$^{\circ }$) corresponds to $\sim$3.6 characters in our layout (or $\sim$1.9 characters at 0.30$^{\circ }$/character, which is more commonly used in standard reading studies). A vertical inaccuracy due to pupil size changes in dark luminance (1.03$^{\circ }$) corresponds to $\sim$0.82 lines in our layout (for comparison, line spacings of this magnitude are common; e.g., the German version of the MECO-corpus uses about 1.28$^{\circ }$).^1^ In other words, under typical reading conditions, inaccuracy due solely to pupil size fluctuations during an experiment with constant luminance can amount to several characters horizontally and more than half a line vertically. If the pupil size during calibration of the eye tracker is not within the range of pupil size during the experimental condition, this can add up to a constant offset (see apparent gaze shift magnitude estimates for between-luminance).

**Fig. 5:**
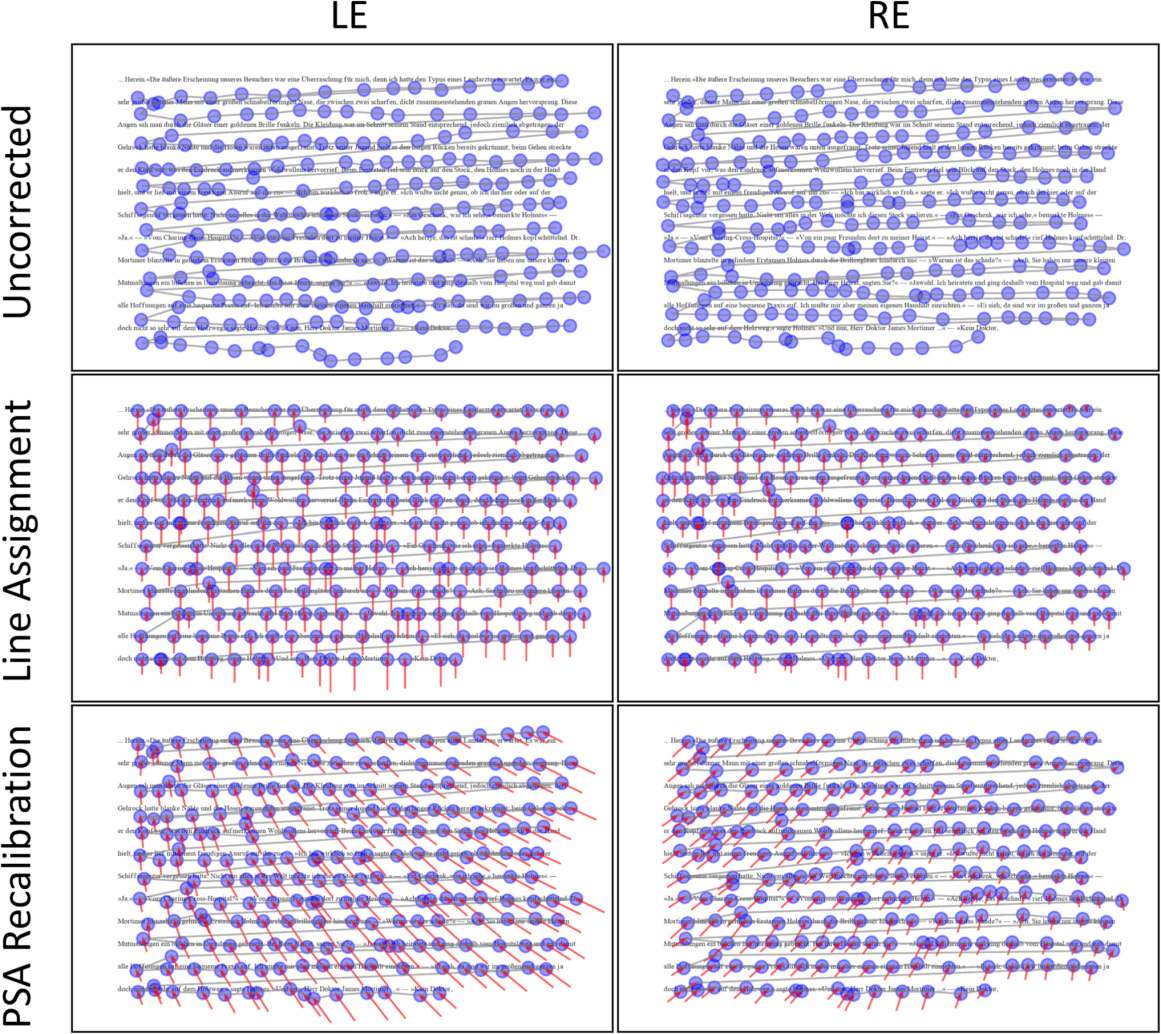
Comparison of corrections applied by line assignment and PSA recalibration on reading data. Reading scanpaths for left eye (LE, *left column*) and right eye (RE, *right column*) from one subject reading a text page under bright luminance conditions. *Blue dots* represent fixation positions, with *red arrows* indicating the corrective vectors applied to each fixation. Note that fixations are used here for visualization purposes, while all metric analyses were conducted on single gaze samples. *Top row*: Uncorrected fixation data showing substantial vertical and horizontal misalignment with the underlying text. *Middle row*: Fixation positions after line assignment, which demonstrate improved vertical alignment but no correction for horizontal offset. *Bottom row*: Fixation positions after PSA recalibration. Note that both methods achieve comparable vertical correction, while only PSA recalibration addresses the contralateral apparent horizontal gaze shift pattern observed in the uncorrected data

### How effective is the PSA recalibration?

To evaluate how well PSA recalibration reduces pupil-size-induced apparent gaze shift, we tested it on the PLR Validation data. To account for potential uneven distributions of pupil sizes (i.e., more gaze samples for certain pupil size ranges), we resampled the data using fixed-width pupil diameter bins of 0.1 mm spanning from the 2.5th to 97.5th percentile. For each bin, we calculated the median gaze offsets (across gaze samples) from the target position.

**Fig. 6:**
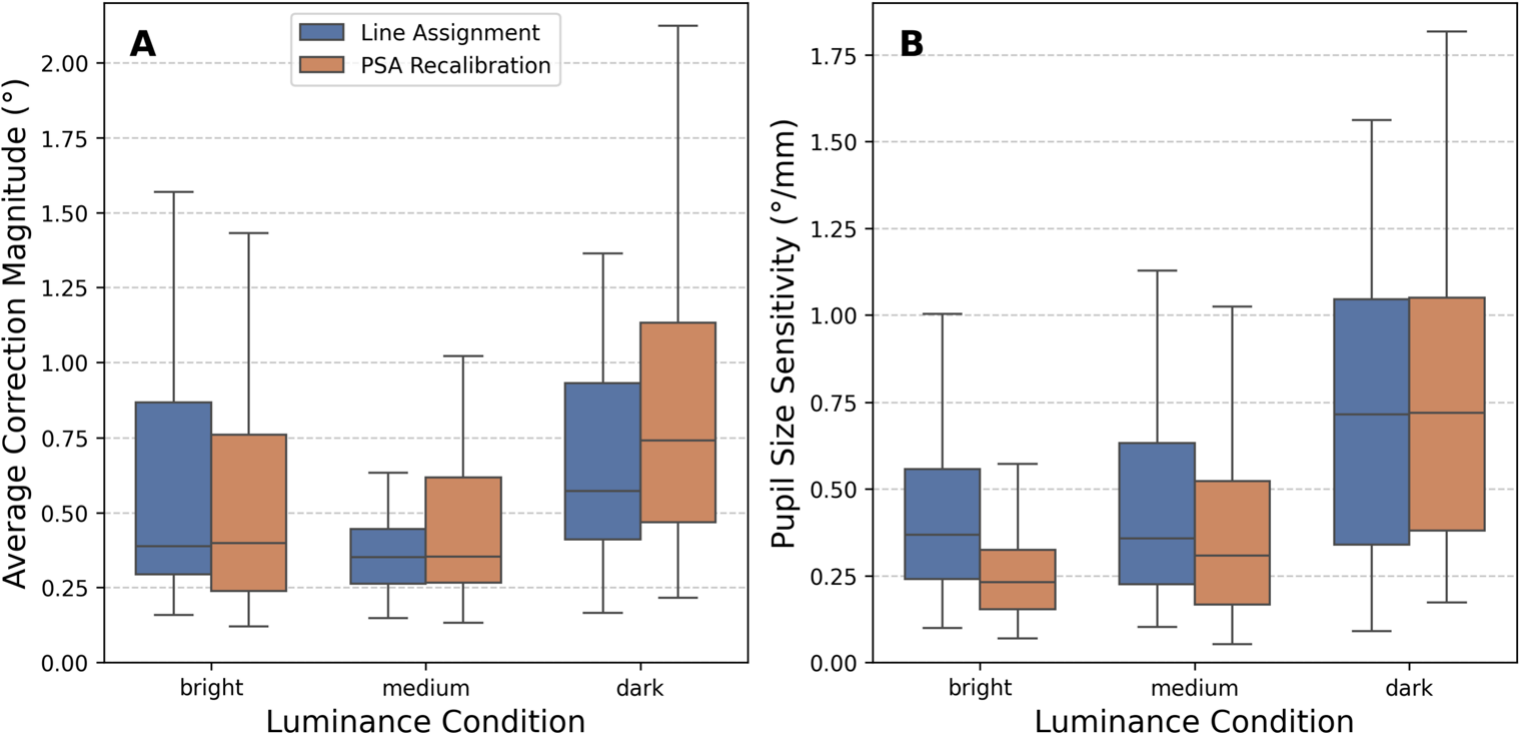
Comparison of vertical correction applied by line assignment (*blue*) and PSA recalibration (*orange*) methods during reading. **A** Average correction magnitude (mean absolute vertical corrective offsets applied to each gaze sample) across all subjects, screen positions (screen divided into a 3$\times$3 grid), and both eyes for each luminance condition. Both methods show similar correction magnitudes, with the lowest magnitudes in the medium compared to both bright and dark luminance conditions. **B** Pupil size sensitivity (slope of vertical corrective offset per millimeter increase in pupil diameter), representing how vertical corrective offsets change with pupil size across all subjects and conditions. Both methods show similar patterns across luminance conditions, with higher pupil size sensitivity in darker conditions. *Box plots* show median and interquartile range across participants, with whiskers extending to 1.5 $\times$ IQR

PSA recalibration substantially reduced pupil-size-induced apparent gaze shift across all validation targets. Figure 4 shows these improvements through two complementary measures:

*Absolute offset reduction:* We quantified how much gaze positions deviated from known target locations. PSA recalibration achieved a median reduction of 50.6% in absolute offsets (IQR 32.3–65.5%), decreasing from 0.78$^{\circ }$ [0.55$^{\circ }$ – 1.06$^{\circ }$] to 0.37$^{\circ }$ [0.27$^{\circ }$ – 0.50$^{\circ }$] overall. Improvements were consistent across dimensions: vertical gaze offsets decreased from 0.47$^{\circ }$ [0.31$^{\circ }$ – 0.73$^{\circ }$] to 0.22$^{\circ }$ [0.15$^{\circ }$ – 0.31$^{\circ }$], and horizontal gaze offsets from 0.45$^{\circ }$ [0.29$^{\circ }$ – 0.65$^{\circ }$] to 0.23$^{\circ }$ [0.16$^{\circ }$ – 0.34$^{\circ }$] (all values median [IQR]).

*Gaze dispersion reduction:* To assess whether the PSA recalibration successfully corrected for pupil-size-dependent variability, we operationalized gaze dispersion across different pupil sizes using the 68% bivariate contour ellipse area (BCEA) calculated across median gaze positions. Since the PLR validation phases systematically induced pupil size changes, this measure directly indicates whether gaze dispersion linked to those changes is reduced. PSA recalibration achieved large improvements in dispersion, with a median reduction of 63.0% (IQR 43.7–74.7%). Median BCEA values decreased from 0.71 $deg^2$ [0.39–1.19 $deg^2$] to 0.25 $deg^2$ [0.14–0.43 $deg^2$].

These results demonstrate that PSA recalibration effectively reduces pupil-size-induced apparent gaze shift, with particularly strong improvements in reducing gaze dispersion. The method addresses both the magnitude of absolute gaze offsets and the variability introduced by pupil size changes across participants.

### Correction of apparent gaze shift during reading

To compare line assignment and PSA recalibration during reading (Research Question 4), we analyzed the vertical corrective offsets applied by each method, examining both their magnitude and their sensitivity to pupil size changes. Analysis was limited to the vertical dimension since line assignment only provides corrections in the vertical dimension (mapping fixations to text lines). Since reading data do not provide known target positions for direct accuracy assessment, we compare the corrections themselves: larger correction magnitudes may indicate greater apparent gaze shift being addressed, while pupil size sensitivity reveals whether corrections systematically vary with pupil diameter – a defining feature of PSA effects.

#### Qualitative comparison of correction patterns

Figure 5 illustrates apparent gaze shift and corrective offsets applied by line assignment and PSA recalibration to fixation scanpaths from one subject. The uncorrected data (top row) shows significant apparent vertical and horizontal gaze shift relative to the text. Apparent vertical gaze shifts from text lines are as large as the distance between text lines and are largely similar between eyes, although slightly smaller for the right eye. Apparent horizontal gaze shift from text boundaries shows a contralateral pattern that varies with screen position (e.g., larger apparent gaze shift for the right eye on the left side of the screen), consistent with the findings in Section Magnitude and individual variability of the PSA.

Line assignment (middle row) effectively eliminates apparent vertical gaze shift by design, but does not correct for apparent horizontal gaze shift. In contrast, PSA recalibration (bottom row) provides vertical corrective offsets similar to those of line assignment, but also reduces apparent horizontal gaze shift, resulting in fixations that are more closely aligned with the underlying text.

This example was chosen for clarity but is representative of the dataset. Participant-wise panels (both eyes, same page, across luminance conditions) showing analogous patterns are provided in our OSF repository (full set for all 30 participants; see Data and Code Availability for the link). While the effectiveness of PSA recalibration varies, the spatial structure and direction of the corrections are consistent.

#### Quantitative comparison of correction magnitude and pupil size sensitivity

To quantify these correction patterns, we calculated two measures for each method. For the correction magnitude, we calculated the mean across all vertical corrective offsets applied to each gaze sample for each subject and condition. For the pupil size sensitivity, we calculated the mean of regression slopes between pupil size and corrective offsets (indicating how much correction changes per millimeter of pupil size change) for each subject and condition. We used absolute values before averaging to account for potentially different directions of the PSA between subjects or screen positions. These measures were analyzed using separate linear mixed-effects models that accounted for random subject variation and tested the influence of correction method, luminance condition, eye, and gaze position.

Figure 6 compares the correction magnitude and pupil size sensitivity between line assignment and PSA recalibration across participants. To account for differences across the screen, we divided the screen into a 3$\times$3 grid and calculated correction magnitude and pupil size sensitivity for each region separately before averaging absolute values across regions.

Both methods applied corrective offsets of broadly comparable magnitude across luminance conditions (Fig. 6A), with median correction magnitudes ranging from 0.35$^{\circ }$ to 0.74$^{\circ }$ across methods and conditions. Notably, both line assignment and PSA recalibration showed the smallest average correction magnitudes in the medium luminance condition compared to bright and dark conditions. Importantly, the EyeLink calibration was performed under medium luminance, which likely contributed to this condition showing the smallest correction magnitude. We will discuss this further in Section The relevance of pupil size during eye tracker calibration. This visual similarity was confirmed by a linear mixed-effects model, which identified a small yet statistically significant difference in correction magnitude between methods ($\beta$ = -0.049$^{\circ }$, *p* = .003), with line assignment applying slightly smaller corrections on average. However, this difference was modest relative to the overall correction magnitudes observed.

Both methods also show clear pupil size sensitivity (Fig. 6B), with median values ranging from 0.23$^{\circ }$/mm to 0.72$^{\circ }$/mm across methods and conditions. Line assignment showed significantly higher sensitivity overall ($\beta$ = 0.065$^{\circ }$/mm, *p* = .002), with stronger effects in bright conditions (interaction: $\beta$ = 0.079$^{\circ }$/mm, *p* = .009) and weaker effects in dark conditions (interaction: $\beta$ = -0.140$^{\circ }$/mm, *p* = < .001) compared to PSA recalibration. However, these differences were relatively small in the context of the overall range of pupil size effects.

As expected from the qualitative comparison, both models showed a significant effect of vertical screen position, with correction magnitudes and pupil size sensitivity decreasing towards the top of the screen (both *p* < .001). A small but significant effect of eye was found for correction magnitude ($\beta$ = -0.054$^{\circ }$, *p* = .001), with slightly smaller corrections applied to the right eye, while its effect on pupil size sensitivity was marginally significant (*p* = .055). These effects align with the asymmetric apparent horizontal and vertical gaze shift patterns described in Section Magnitude and individual variability of the PSA.

Between-subject variability explained a substantial proportion of variance in both correction magnitude (ICC = 0.41) and pupil size sensitivity (ICC = 0.33), underscoring the importance of accounting for individual differences in gaze behavior and susceptibility to PSA effects.

Taken together, these results indicate that while minor statistical differences exist between line assignment and PSA recalibration, both methods apply corrections of broadly comparable magnitude and exhibit similar sensitivity to pupil size changes during reading. These findings suggest that although line assignment does not explicitly model pupil size, it implicitly captures pupil-size-induced apparent gaze shift to a degree comparable with dedicated PSA recalibration in typical reading scenarios.

## Discussion

In this study, we investigated the impact of changes in pupil size on eye tracking accuracy during reading tasks. Our focus was motivated by the surprising gap in reading research regarding the PSA – a systematic source of spatial inaccuracy that occurs when pupil dilation or constriction causes apparent shifts in gaze position. Despite the high demands for spatial accuracy in reading research and the known susceptibility of video-based eye trackers to this effect, the PSA has received little attention in reading contexts. We sought to characterize the PSA in our setup, examine pupil size changes during luminance-controlled reading, and compare two correction approaches: line assignment (a standard method commonly used in reading research) and PSA recalibration (a specific correction method previously shown to reduce PSA in non-reading contexts).

### Key findings

Our study revealed substantial PSA effects, with apparent gaze shift reaching up to 2$^{\circ }$ as pupil size changed from 2 to 6 mm. Horizontal PSA showed systematic contralateral patterns (apparent gaze shift occurred when the target and measured eye were on opposite sides) with median slopes for horizontal apparent gaze shift of 0.38$^{\circ }$/mm. PSA slopes for vertical apparent gaze shift were larger for larger pupil sizes, increasing to a median of 0.86$^{\circ }$/mm for 5–6 mm pupils, and were more pronounced at the bottom of the screen. These effects were mirrored between eyes but with slight asymmetries, particularly visible in the right eye, showing smaller vertical shifts.

Pupil sizes varied substantially during reading, even within a constant luminance condition. While median pupil size increased from 2.89 mm (bright) to 5.32 mm (dark), more critically, pupil size changed by 0.78–1.38 mm within luminance conditions. These pupil size changes occurred continuously, including within single pages. Notably, bright conditions showed not only smaller pupil sizes but also reduced within-subject and between-subject variability, suggesting more consistent measurement conditions both within and across participants.

Translating PSA slopes and observed pupil-size changes into estimated apparent gaze shift magnitudes during reading, average apparent gaze shift magnitudes within luminance conditions were 0.39$^{\circ }$ – 0.64$^{\circ }$ horizontally and 0.24$^{\circ }$,– 1.03$^{\circ }$ vertically across luminance conditions (Table 1). Between-luminance estimates were of similar order (0.49$^{\circ }$ – 0.66$^{\circ }$). Using a typical character width of $\approx {0.3}{^{\circ }}$ in reading studies, a horizontal error of 0.58$^{\circ }$ corresponds to $\sim$2 characters. If the calibration pupil size falls outside the pupil size range observed during the experimental condition, this can add up to a constant offset (see between-luminance estimates). Given the substantial individual differences and dependencies on screen position and eye, these effects could be considerably larger. Consistent spatial patterns (contralateral horizontal; stronger vertical at the bottom) and eye-specific asymmetries further modulate the impact of pupil size changes on apparent gaze shift.

Our comparison of correction methods revealed that line assignment implicitly compensates for vertical PSA effects. Both line assignment and PSA recalibration applied similar vertical correction magnitudes and showed similar pupil size sensitivities. However, while both methods effectively corrected vertical shifts, only PSA recalibration addressed apparent horizontal gaze shift, reducing median absolute gaze offset and gaze dispersion on independent PLR validation data by 50.6% and 63.0%, respectively.

Throughout our analyses, we found substantial individual differences in PSA effects, with subject-level random effects accounting for 33–41% of variance in corrections applied by both methods. This highlights that PSA is not only a systematic error but also highly idiosyncratic, requiring individualized approaches for optimal correction.

While both correction methods achieve broadly comparable vertical correction magnitudes, they represent fundamentally different approaches to addressing gaze inaccuracy. Line assignment is primarily a heuristic method that relies on assumptions about typical reading behavior (i.e., that fixations predominantly target text lines) and often incorporates subjective manual adjustments. In contrast, PSA recalibration represents an analytical approach based on empirically measured relationships between pupil size and apparent gaze shift. The choice between these methods involves trade-offs: line assignment leverages domain-specific knowledge about reading patterns but may be limited by its assumptions, while PSA recalibration provides a data-driven correction but requires additional calibration procedures.

### Algorithmic line assignment: Comparison with PSA recalibration

We complemented the main analysis by repeating the comparison of applied corrections of our supervised line assignment and PSA recalibration, and their relation to pupil size, with automatic line assignment algorithms. We do so because automatic line assignment is widely used in multi-line reading studies (e.g., Carr et al., 2022; Glandorf and Schroeder, 2021), and our supervised line assignment with human verification likely represents a best-case scenario or manual correction. We use two commonly used recent algorithms: “Slice” (Glandorf & Schroeder, 2021) and “Warp” (Carr et al., 2022). We additionally examined whether pre-correcting fixations with PSA recalibration improves these algorithms’ performance (for completeness, we also tested the transformer-based DIST (Mercier et al., 2024), but on our data it consistently ignored the last text line; dataset-specific training would likely be required, so we do not include it here).

We re-ran the same linear mixed-effects models – separately for vertical correction magnitude and pupil-size sensitivity as dependent variables – including “Slice” and “Warp” as additional correction methods (model structure remains unchanged).

Relative to supervised line assignment, PSA recalibration – as already shown in the main analysis – applied slightly larger vertical corrections ($\beta$ = 0.054$^{\circ }$, $p=.0019$), “Warp” slightly smaller ($\beta$ = -0.087$^{\circ }$, $p<.001$), and “Slice” markedly larger ($\beta$ = 0.354$^{\circ }$, $p<.001$). For pupil-size sensitivity, main-effect differences were modest, with luminance interactions paralleling the main analysis: in bright luminance, “Slice” and “Warp” were somewhat more sensitive than supervised line assignment ($\beta$ = 0.114 and 0.101$^{\circ }$/mm; $p=.0066$ and .0156), whereas in dark luminance, PSA recalibration showed higher sensitivity ($\beta$ = 0.143$^{\circ }$/mm, $p=.0012$). Taken together, these patterns indicate that both automatic algorithms apply vertical corrections broadly comparable to our supervised line assignment and PSA recalibration, both in terms of absolute magnitude and sensitivity to pupil size changes – i.e., this suggests that they can compensate for vertical PSA. At the same time, the markedly larger correction magnitudes for “Slice” point to a different correction behavior (more aggressive vertical shifts), which we revisit below when considering fixation-to-line agreement.

**Table 2:** Agreement of fixation-to-line assignment with our supervised line assignment across (overall) and by luminance condition

Method	Overall	Bright	Medium	Dark
Attach	69.1%	66.2%	81.0%	57.7%
PSA recalibration+Attach	83.0%	93.9%	83.4%	69.1%
Slice	77.9%	87.8%	74.3%	70.8%
PSA recalibration+Slice	80.5%	91.7%	79.4%	68.4%
Warp	91.3%	88.0%	94.2%	91.4%
PSA recalibration+Warp	96.9%	98.1%	97.8%	94.4%

Uncorrected fixations and fixations corrected by PSA recalibration were attached to the nearest line (“attach”; Carr et al., 2022)

To give a more complete picture of the performance of the line assignment algorithms and to see whether performance improved when pre-correcting with PSA recalibration, we assessed fixation-to-line agreement (percent agreement with supervised line assignment) for the correction methods. For methods that do not themselves perform line assignment (uncorrected data and PSA recalibration), we used the simplest assignment rule by attaching each fixation to its nearest text line (“attach”; Carr et al., 2022). This gives us six different correction methods that are compared against our supervised line assignment: “attach”, PSA recalibration+“attach”, “Slice”, PSA recalibration+“Slice”, “Warp”, and PSA recalibration+“Warp”.

Table 2 shows the fixation-to-line agreement for each of these methods. PSA recalibration alone improved agreement over attach (overall: from $69.1\%$ to $83.0\%$). “Warp” performed well ($91.3\%$) and benefitted further from PSA pre-correction ($96.9\%$ overall; Bright $98.1\%$, Medium $97.8\%$, Dark $94.4\%$). “Slice” improved with PSA recalibration (from $77.9\%$ without pre-correction to $80.5\%$), though agreement remained lower – especially in medium and dark luminance. This accords with the mixed-model results: “Warp”, whose correction magnitudes were closest to supervised line assignment and PSA recalibration also achieved the highest fixation-to-line agreement and benefited most from PSA pre-correction, whereas “Slice” – which showed markedly larger magnitudes – agreed less in the fixation-to-line assignment with supervised line assignment (and with “Warp”/PSA recalibration).

Taken together, addressing the PSA with PSA recalibration improves the performance of line assignment algorithms. Among tested options, PSA recalibration+“Warp” most closely matched supervised line assignment across luminance conditions, while PSA recalibration alone already outperformed “Slice” in agreement. While these additional analyses were not part of our predefined research questions, they provide a practical guide: applying PSA recalibration prior to line assignment can improve the robustness for automated line assignment pipelines.

### The relevance of pupil size during eye tracker calibration

The larger average correction magnitude we observed in both bright and dark conditions (compared to medium) point to a critical insight: a substantial portion of gaze inaccuracy likely results from pupil size differences between eye tracker calibration and actual reading conditions. Previous research has indicated that systematic differences in pupil size between calibration and experimental phases can introduce significant biases in measured gaze position (Jaschinski, 2016). This effect has also been demonstrated by Huckauf (2018), who found reduced fixation disparity (horizontal difference between left and right eye gaze signals) when using consistent background conditions across calibration and reading phases compared to when different backgrounds were used. Consistent with this interpretation, we estimated apparent gaze shift magnitudes of 0.49$^{\circ }$ – 0.66$^{\circ }$ between luminance conditions (Table 1), that arise solely from baseline pupil-size differences between reading conditions using different background luminance. Given that we calibrated under medium luminance, the larger average correction offset in bright and dark conditions likely reflect the mismatch between calibration and reading pupil sizes.

Both line assignment and PSA recalibration effectively compensated for these calibration-related offsets. However, our implementation of line assignment represents a rather ideal scenario involving semi-automated processing with manual supervision. Pure algorithmic approaches to line assignment may struggle with very large offsets, as noted by Carr et al. (2022) and suggested by the results of the “Slice” algorithm in the previous section. Our findings and previous findings suggest that bright backgrounds during both calibration and reading may provide advantages for eye-tracking accuracy, as they produce smaller pupils with a smaller range and smaller PSA slopes for vertical apparent gaze shift (Drewes et al., 2014). As shown in Section 4.2 (Table 2), pre-correcting with PSA recalibration improves algorithmic line assignment and is therefore a promising approach: first apply PSA recalibration to reduce systematic inaccuracy from pupil-size changes (in both horizontal and vertical directions), then follow with algorithmic line assignment to refine any remaining vertical offsets to text lines.

### Strategies for addressing PSA in reading studies with pupil-based eye trackers

Our findings on the magnitude of PSA effects and pupil size changes during reading highlight the importance of addressing these artifacts in research that requires high eye-tracking data accuracy. While no single approach provides a complete solution for all experimental contexts, several correction strategies are available, each with specific advantages and limitations. These include established eye-tracking practices (eye-tracker recalibration, one-point drift correction, line assignment) as well as dedicated PSA correction methods.

*Eye-tracker recalibration* offers a straightforward app-roach to correct systematic pupil size changes that occur over longer timescales, e.g., change in arousal state and cognitive demands (Choe et al., 2016). It can potentially reduce the overall apparent gaze shift magnitude due to the PSA. It could also account for gaze position and eye-dependent PSA effects, and runs online. However, it cannot address pupil size changes on smaller timescales (within pages), and recalibration between each reading page may be impractical, since it might disrupt the reading flow.

*One-point drift correction*, commonly implemented in commercial eye-tracking systems like the EyeLink, provides a simpler alternative that requires minimal participant engagement and may effectively address systematic offsets from pupil size differences between calibration and reading conditions or gradual pupil size changes over time. It can be implemented online as well as with offline processing. However, our finding of position-dependent PSA patterns suggests this approach has limitations. Given that we observed substantial differences in PSA effects across different screen positions, a correction applied at a single point would inadequately address the apparent gaze shift occurring elsewhere on the screen, particularly in the horizontal dimension and in peripheral regions.

*Line assignment*, widely used in reading research, effectively addresses vertical PSA components without explicitly modeling pupil size changes. Our results indicate that these methods implicitly capture apparent vertical gaze shift related to pupil size changes. However, they leave horizontal shifts unaddressed, which is particularly problematic for research questions requiring accurate horizontal gaze measurements. Furthermore, large vertical offsets can impair the performance of automated line assignment algorithms, often necessitating manual intervention. Importantly, line assignment – whether performed automatically or manually – can also be complicated when participants skip lines or do not read sequentially, making reliable gaze-to-line mapping more difficult. Most line assignment approaches will only run offline, since they rely on a longer sequence of fixations rather than single fixations or gaze samples.

*PSA recalibration* by inducing pupil-size changes in a dedicated calibration procedure provides the most comprehensive correction by addressing both horizontal and vertical components of gaze inaccuracy. This approach accounts for the idiosyncratic nature of PSA and its spatial distribution, offering superior correction for position-dependent and eye-specific effects. While our current approach was used in an offline analysis, this approach could also be used online, since the recalibration runs on the gaze sample level and is fast enough to be used online, once the PSA mapping function has been fit. The primary limitation is the requirement for a dedicated calibration procedure, which takes additional time – here we used two PLR phases, each taking about 2 min – and requires participants to reliably fixate the targets. Additionally, for optimal results, the pupil size range during PSA recalibration should cover the pupil size range during experimental conditions.

### Choosing the right approach

The selection of an appropriate correction strategy ultimately depends on the specific research questions and experimental constraints. For multi-line studies primarily concerned with which line is being read, conventional line assignment and/or one-point drift correction may suffice. However, research examining precise landing positions within words or similar fine-grained horizontal measures would benefit significantly from PSA recalibration or a combined approach that first applies PSA recalibration to minimize systematic pupil-size influence, followed by line assignment for final refinement.

### Implications and recommendations for reading researchers

Based on our findings, previous literature, and established eye tracking practices, we offer the following practical recommendations for reading researchers using video-based eye trackers that rely on pupil center detection to improve gaze data accuracy by addressing the PSA:

#### Awareness and documentation

 

- *Acknowledge PSA as a significant source of error:* Recognize that pupil-size-induced apparent gaze shift can be substantial and varies systematically with pupil size, even in luminance-controlled experiments.
- *Report pupil size metrics:* Include pupil size statistics (means and range) in reading studies, even when pupil size is not the primary variable of interest. This documentation allows readers to assess potential PSA influences and supports cross-study comparisons. Where possible, check whether pupil diameter systematically covaries with the variables of interest, e.g., model pupil diameter as a covariate in statistical analyses.
- *Account for individual differences:* Be aware that considerable between-subject differences in both pupil size dynamics and PSA slopes can result in significant variability in gaze accuracy, even under identical experimental conditions.

#### Experimental design considerations

 

- *Use bright backgrounds:* Whenever possible, use bright screen backgrounds for reading stimuli, following the principle that “smaller is better” for pupil-based eye tracking (Drewes et al., 2014). Our findings highlight three advantages of bright conditions: (1) smaller pupil sizes, reducing PSA slopes for vertical apparent gaze shift; (2) smaller pupil size ranges during reading; and (3) reduced inter-subject variability, improving accuracy consistency across participants.
- *Match calibration and reading conditions:* Calibrate the eye tracker under the same luminance conditions as the reading task to minimize systematic offsets between calibration and experimental conditions. Keep in mind that pupil size may still change over time due to factors unrelated to screen brightness (e.g., fatigue, cognitive load, or adaptation), even when luminance remains constant.
- *Adapt setup and stimulus positioning based on PSA patterns:* Use known PSA characteristics (e.g., contralateral effects, stronger vertical PSA at the screen bottom for the EyeLink system) to guide decisions about which eye to track and where to place critical stimuli. Whether and how to adapt may depend on your research question, apparatus, and stimulus material, as these factors influence both the manifestation of PSA patterns and the feasibility of adjustments.
- *Adapt stimulus design:* In general, inaccuracy can be add-ressed through stimulus design, such as larger text size, inc-reased line spacing, and larger areas of interest. However, be aware that these adjustments may influence gaze behavior (Chiu & Drieghe, 2023; Slattery & Rayner, 2013). Studies that use single-line designs will also not be (or less) affected by the vertical inaccuracy due to the PSA.

#### Correction strategies

 

- *Choose appropriate correction methods:* Match correction approaches to your measurement requirements and practical constraints, considering factors such as whether you need vertical-only or both vertical and horizontal corrections, your willingness to perform manual work, whether you need online correction, and whether you can accept certain assumptions about gaze patterns.
- *Consider temporal dynamics of the PSA:* Since pupil size may change continuously, even during fixations Choe et al. (2016), consider correcting for the PSA at gaze-sample level rather than the fixation level. Note that longer fixations may accumulate more pupil-size-induced apparent gaze shift – a potential confound with cognitive processing effects that warrants future investigation.

## Limitations and future directions

### Generalizability across setups

PSA patterns depend on viewing geometry, camera placement, and tracker specifics. Our mapping was obtained with an EyeLink 1000 Plus (desktop mode; slightly angled camera). The observed spatial pattern for the PSA can be different for other eye trackers, as shown by Hooge et al. (2021).

### Robustness to unstable fixation

Future studies should quantify how deviations from stable fixation (e.g., drift or micro-saccades) influence PSA recalibration and how the robustness of the method can be increased if needed – e.g., by discar-ding detected micro-/saccades during fitting or by using constant backgrounds across phases (Drewes et al., 2014), thereby reducing the need for prolonged fixation. Ideally, such validation would employ a reference eye tracker that is largely insensitive to PSA (e.g., retinal imaging, dual-Purkinje image, or scleral-coil systems; cf. Hooge et al., 2025). In addition, it could be investigated whether the accuracy and robustness of PSA recalibration differ when it is performed separately for each eye (i.e., monocular calibration).

### Population considerations

Some groups may exhibit reduced fixation stability (e.g., school-age children; cf. Serpa et al., 2023); thus, PSA recalibration may be less feasible there. Shorter fixation epochs, larger or optimized fixation targets, more frequent breaks, or alternative procedures for estimating PSA slopes may be required.

### Typography and stimulus dependence

Our reading layout (a proportional font with on average 0.16$^{\circ }$/character) differs from many reading study designs (often using monospace fonts with about 0.3$^{\circ }$/character). While we did not aim to replicate word-level effects, future studies should examine different fonts, word lengths, and visual densities, and verify whether PSA-related apparent gaze shift magnitudes translate proportionally across layouts.

### Word-level reading effects

We did not analyze downstream word-level metrics (e.g., word frequency, landing positions, skipping, refixations). Whether and how PSA influences – or even biases – such effects will depend on layout, analytic choices, and any co-variation of pupil size with lexical variables. We encourage future work to quantify whether and how PSA – and its correction – affects these metrics.

## Conclusion

The PSA remains an under-addressed source of spatial error in reading research. Our study confirms that substantial, systematic apparent gaze shifts occur even under controlled luminance, reducing both vertical and horizontal gaze accuracy. We demonstrated that pupil size changes during reading can induce apparent gaze shift which could potentially have significant practical implications for key reading metrics including landing positions, word skipping, and line assignment accuracy. This potential calls for further future work investigating the influence of the PSA on specific reading effects.

PSA recalibration offers an effective solution for addressing these systematic errors, substantially reducing both vertical and horizontal inaccuracies through an analytical approach based on empirically measured pupil-size-dependent relationships. Our validation demonstrated substantial improvements in both absolute gaze accuracy and pupil-size-dependent gaze dispersion.

Importantly, we provide the first evidence that line assignment procedures implicitly correct for vertical PSA effects, despite not modeling pupil size directly. However, horizontal PSA effects remain unaddressed by line assignment alone. Dedicated PSA recalibration offers a more comprehensive analytical solution that addresses both vertical and horizontal inaccuracies through empirically measured pupil-size-dependent relationships rather than behavioral assumptions. Additionally, we show that the performance of line assignment algorithms can increase, when pre-correcting by PSA recalibration.

Given the accuracy demands in reading research and the substantial individual differences we observed, we recommend that pupil size changes, and, as a result, inaccuracy due to the PSA are routinely considered in study design, reporting, and data correction workflows. The idiosyncratic nature of PSA underscores the importance of empirical approaches to gaze correction rather than relying solely on behavioral assumptions.

## Supplementary Information

Below is the link to the electronic supplementary material.

Supplementary file 1 (pdf 102 KB)

## Data Availability

Aggregated data, reading stimuli and raw gaze data from one consenting participant is publicly available with the code at https://osf.io/uzej3. Full raw gaze data unavailable due to privacy regulations, but are available from the corresponding author on reasonable request.

## Footnotes

1 Computed from the German MECO parameters ($1920\times 1080$ px, $544\times 303$ mm, viewing distance 60 cm; line spacing 48 px) using the SR Research visual-angle calculator; parameters from Glandorf and Schroeder (2021); Siegelman et al. (2022).

## Code Availability

Python and R code for the analyses in this article is available at https://osf.io/uzej3.
