# Supplementary material for: The impact of the pupil size artifact on pupil-based eye-tracking data in reading tasks: Assessment and compensation
Source: Behav Res Methods. 2025 Dec 19;58(1):27. doi: 10.3758/s13428-025-02912-y (PMC12717228; doi:10.3758/s13428-025-02912-y)
Supplement: Supplementary file 1 — (pdf 102 KB) [file 13428_2025_2912_MOESM1_ESM.pdf]

Supplementary Appendix for: The Impact of the Pupil Size Artifact on Pupil-Based Eye Tracking Data in Reading Tasks: Assessment and Compensation.

Appendix A Detailed estimation of PSA during reading across the screen and eyes

Table A1 Estimated within-luminance mean absolute apparent gaze shift magnitude (in °) for the **horizontal** dimension, grouped by eye and horizontal screen region. For each subject and cell, PSA magnitude was computed as $|\text{PSA slope}| \times |\Delta\text{pupil}|$, where the PSA slope (°/mm) is the mean slope of the LOWESS-based PSA curve across the subject's pupil-size range observed within the luminance condition (2.5th–97.5th percentiles), and Δpupil is that per-subject 95% range under constant luminance. Entries are averages across subjects (mean of absolute values). Screen regions correspond to $x \in \{-13.54°, 0°, +13.54°\}$ relative to center (viewer's left/center/right).

Eye	Screen Region	Bright	Medium	Dark
Left	Left	0.13°	0.33°	0.45°
Left	Center	0.36°	0.57°	0.75°
Left	Right	0.79°	1.17°	1.36°
Right	Left	0.40°	0.68°	0.78°
Right	Center	0.16°	0.27°	0.36°
Right	Right	0.43°	0.45°	0.49°

Table A2 Estimated within-luminance mean absolute apparent gaze shift magnitude (in °) for the **vertical** dimension, grouped by eye and vertical screen region. Computation matches Table A1: per-subject |PSA slope| × |Δpupil| with slopes averaged over the subject's observed pupil-size range within the luminance condition and Δpupil as the per-subject 95% range; values are then averaged across subjects (mean of absolutes). Screen regions correspond to $y \in \{-6.4°, 0°, +6.4°\}$ relative to center (bottom/center/top; negative y = bottom).

Eye	Screen Region	Bright	Medium	Dark
Left	Bottom	0.37°	0.84°	1.30°
Left	Center	0.18°	0.68°	1.04°
Left	Top	0.16°	0.54°	0.93°
Right	Bottom	0.33°	0.85°	1.33°
Right	Center	0.19°	0.68°	1.05°
Right	Top	0.18°	0.53°	0.63°

Table A3 Estimated between-luminance mean absolute PSA magnitude (in °) for the **horizontal** dimension, grouped by eye and horizontal screen region. For each subject and cell, PSA magnitude was computed as |PSA slope| × |Δpupil|, where Δpupil is the per-subject difference in median pupil diameter between luminance conditions (Bright→Medium, Medium→Dark). PSA slopes (°/mm) are the mean slopes of the LOWESS-based PSA curves across the pupil-size span between those medians. Entries are averages across subjects (mean of absolute values). Screen regions correspond to $x \in \{-13.54°, 0°, +13.54°\}$ (viewer's left/center/right).

Eye	Screen Region	Bright→Medium	Medium→Dark
Left	Left	0.24°	0.35°
Left	Center	0.45°	0.55°
Left	Right	1.05°	0.99°
Right	Left	0.55°	0.55°
Right	Center	0.24°	0.24°
Right	Right	0.47°	0.28°

Table A4 Estimated between-luminance mean absolute PSA magnitude (in °) for the **vertical** dimension, grouped by eye and vertical screen region. Computation as in Table A3: per-subject |PSA slope| × |Δpupil| with Δpupil the difference in median pupil diameter between luminance conditions and slopes averaged over the pupil-size span between those medians; values are averaged across subjects (mean of absolutes). Screen regions correspond to $y \in \{-6.4°, 0°, +6.4°\}$ (bottom/center/top; negative y = bottom).

Eye	Screen Region	Bright→Medium	Medium→Dark
Left	Bottom	0.70°	0.88°
Left	Center	0.48°	0.64°
Left	Top	0.36°	0.54°
Right	Bottom	0.60°	0.84°
Right	Center	0.46°	0.66°
Right	Top	0.40°	0.45°
